# Computable visually observed phenotype ontological framework for plants

**DOI:** 10.1186/1471-2105-12-260

**Published:** 2011-06-24

**Authors:** Jaturon Harnsomburana, Jason M Green, Adrian S Barb, Mary Schaeffer, Leszek Vincent, Chi-Ren Shyu

**Affiliations:** 1 Department of Computer Science, University of Missouri, 201 EBW, Columbia, MO, 65211, USA; 2 Informatics Institute, University of Missouri, 241 EBW, Columbia, MO, 65211, USA; 3 Division of Plant Sciences, University of Missouri, 1-41 Agriculture Building, Columbia, MO, 65211, USA; 4 Information Science Department, School of Graduate Professional Studies, Pennsylvania State University - Great Valley, 30 E Swedesford Rd, Malvern, PA, 19355, USA; 5 USDA-ARS, Plant Genetics Research Unit, 203 Curtis Hall, Columbia, MO, 65211, USA

## Abstract

**Background:**

The ability to search for and precisely compare similar phenotypic appearances within and across species has vast potential in plant science and genetic research. The difficulty in doing so lies in the fact that many visual phenotypic data, especially visually observed phenotypes that often times cannot be directly measured quantitatively, are in the form of text annotations, and these descriptions are plagued by semantic ambiguity, heterogeneity, and low granularity. Though several bio-ontologies have been developed to standardize phenotypic (and genotypic) information and permit comparisons across species, these semantic issues persist and prevent precise analysis and retrieval of information. A framework suitable for the modeling and analysis of precise computable representations of such phenotypic appearances is needed.

**Results:**

We have developed a new framework called the Computable Visually Observed Phenotype Ontological Framework for plants. This work provides a novel quantitative view of descriptions of plant phenotypes that leverages existing bio-ontologies and utilizes a computational approach to capture and represent domain knowledge in a machine-interpretable form. This is accomplished by means of a robust and accurate semantic mapping module that automatically maps high-level semantics to low-level measurements computed from phenotype imagery. The framework was applied to two different plant species with semantic rules mined and an ontology constructed. Rule quality was evaluated and showed high quality rules for most semantics. This framework also facilitates automatic annotation of phenotype images and can be adopted by different plant communities to aid in their research.

**Conclusions:**

The Computable Visually Observed Phenotype Ontological Framework for plants has been developed for more efficient and accurate management of visually observed phenotypes, which play a significant role in plant genomics research. The uniqueness of this framework is its ability to bridge the knowledge of informaticians and plant science researchers by translating descriptions of visually observed phenotypes into standardized, machine-understandable representations, thus enabling the development of advanced information retrieval and phenotype annotation analysis tools for the plant science community.

## Background

In recent years, the biology community has been increasingly focused on locating genes and identifying their functions. Advances in technology have allowed a vast amount of biological data to be collected, including the sequencing and annotation of rapidly growing numbers of genomes, the generation of genetic and physical maps, and the description of both mutant and wild type phenotypes/traits. Each data collection, however, has its own scope, terminology, and descriptions, which vary not only across domains but also within domains by research group and individual. To unify the vocabulary and descriptions within a domain, and also to serve as a bridge among various sub-domains, many bio-ontologies have been developed. In fact, there has been enormous effort put forth by the biology community towards the development of various ontologies [1-8] with a significant contribution being made thereby to plant science research. For example, the Gene Ontology (GO) [9,10] contains terms and definitions used to describe biological processes, cellular components, and molecular functions. This ontology has been developed to allow ready access to gene function knowledge across different species. Other relevant ontology examples include the Plant Ontology (PO) [11-13], an umbrella ontology covering various sub-domains in the plant realm including growth/developmental stages, plant structure [8], and the Trait Ontology (TO), a sub-ontology that describes characteristics of a plant such as height, leaf color, and disease resistance [14-17]; and the Phenotype and Trait Ontology (PATO), which is an ontology of phenotypic qualities defined "to be used in conjunction with ontologies of 'quality-bearing entities'" [18]. PATO contains descriptions of many general phenotypic qualities, including both qualitative and quantitative characteristics, and some groups have expressed interest in using PATO for phenotype descriptions [19,20].

Phenotype annotations are often recorded based on the Entity-Quality (EQ) model, in which the entity (or trait, in this case) is defined in terms of concepts from one or more ontologies, e.g. PO or GO, and the quality (or value of the trait) is assigned to a concept from an ontology of qualities, e.g. PATO. As an example, consider the representation of a "green maize leaf" using the EQ model. One would have to specify the taxon (maize, NCBI Taxonomy ID: 381124), the PO plant structure (leaf, PO:0009025), the TO trait (leaf color, TO:0000299), and the PATO identifier (green, PATO:00003-20). Annotations recorded in this way are said to facilitate comparisons of phenotype descriptions within and across species. By manually translating free-text phenotype descriptions to the EQ model, Washington [21] demonstrated the ability to compare human and animal disease phenotypes utilizing the hierarchical structure of the ontology as well as the annotation frequency. A similar approach for comparing phenotype annotations within and across plant structure and species could be applied.

Because phenotype annotation and curation remain very time-consuming tasks, various tools have been created to help with annotation. Phenote [22] is one such tool that describes phenotypes using the EQ model, and this tool was utilized by Washington in [21]. In addition, the *Solanaceae *community has developed and implemented a user-friendly annotation utility that exploits bio-ontologies [23]. Even with the help of annotating software, it is still overwhelming for curators to annotate all the available phenotype images. Automatic annotation methods are needed, and there has been some work on this topic already. Beck [24] successfully mapped the output from two phenotyping pipelines in mice to both the Mammalian Phenotype ontology as well as the EQ model, which facilitated automatic annotation of mouse phenotype data generated using these pipelines. The approach mainly relies on standardized and measurable phenotypes of mice, such as body weight and average pulse rate, which is different from the subjective descriptions often used to annotate plant phenotypes, such as "small irregular chlorotic lesions on a maize leaf."

**Figure 1:**
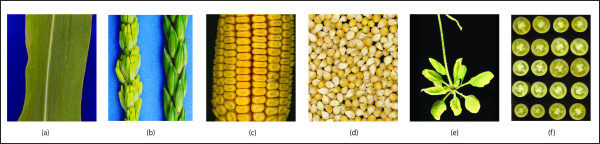
**Example variety of "yellow" phenotypic appearances**. When describing color, annotators tend to use soft, qualitative semantics, e.g. "yellow"; however, these contain significant semantic heterogeneity, as can be seen in the examples of (a) a yellowish corn leaf, (b) a yellow versus green corn tassel (courtesy of MaizeGDB), (c) a yellow corn cob, (d) yellow kernels, (e) a yellowish *Arabidopsis *leaf (courtesy of TAIR), and (f) yellow tomatoes (courtesy of SGN).

Although textual descriptions of phenotypes seem to be clear to human understanding, they are subject to various semantic issues when handled in a computational manner. Despite the tremendous amount of time and effort put forth in their construction and maintenance, current ontologies remain plagued by issues related to semantic *ambiguity*, *granularity*, and *heterogeneity*. *Ambiguity *occurs when a single term has different meanings, though sometimes the differences are very subtle. Consider the various yellow plant structures in Figure 1. One would like the term "yellow" to represent the same quantitative hue across all phenotypes, body parts, and taxa. However, the "yellow" describing a maize leaf (Figure 1 (a)) is obviously different from that which describes a maize ear, kernel, or tassel (Figure 1 (b, c, d)), and the differences are even more striking when one ventures into other taxa (Figure 1 (a, e, f)). Though detailed color names do exist for these yellow variations, phenotype annotators typically use very coarse descriptors for colors, like "yellow", as a soft qualitative description, even though these terms lack the specificity of more specific color names or the rigor of quantitative color measures.

*Granularity *refers to the level of detail used in the definition of an ontological term. With regard to visually observed phenotypes, the terms present in existing ontologies have an insufficient amount of detail to provide precise phenotypic descriptions. For example, the description of a necrotic lesion can be coarsely described in terms of color as "brown". Also, leaf color, as used in PATO, is considered coarse because no guidelines exist for differentiating subtle differences in color, which may be biologically significant, e.g. "green", "light green", and "lemon green".

*Heterogeneity *refers to the situation where the same visually observed phenotype is described with different terms. For example, "yellowish" and "yellow" may be used to describe the same color. A less obvious form of heterogeneity arises when individual image curators have different representations of the same semantics based on their level of expertise and individual specialties [25]; a "green" leaf for one expert may be "light green" for another, or one curator might describe viviparous kernels as "lemon yellow" while another might use "light yellow."

It is not difficult to see the complexity of describing visually observed phenotypes when trying to minimize these semantic issues. Therefore, an ontological framework has been developed for standardizing visual phenotypic information and making that information readily exchangeable within the plant science community. Our proposed framework, the **C**omputable **V**isually **O**bserved **P**henotype **O**ntological **F**ramework (CVOPOF) for plants, not only attempts to solve the abovementioned problems, but also aims to facilitate automatic phenotype image annotation and to improve the precision and accuracy of visually observed phenotype searches. The purpose of this framework is not to develop a complete ontology for any species; instead, the goal is to provide a skeleton for the plant community to follow when defining their traits of interest. Two real-world case studies are included to demonstrate the use of the framework.

## Methods

### Quantitative View of Descriptions of Visually Observed Phenotypes

In this section, we introduce textual annotations of visually observed phenotypes using examples from *Zea mays *(maize) for two purposes. First, we would like to identify the prevailing characteristics described in these annotations to ensure that these are modeled by our framework. Second, and more importantly, we would like to demonstrate the capability and necessity of using computational algorithms for accurate, objective, and comprehensive measurement of these characteristics.

The sample annotations for maize are taken from two sources: (1) Neuffer's Mutants of Maize [26], which provides descriptions of several lesion mimic mutants, and (2) descriptions of Southern Leaf Blight (SLB) infections, based on the scoring rubric mentioned in [27]. Though these sample annotations clearly do not cover every aspect of every known plant phenotype, they do illustrate how the framework can be applied and imply how it can be extended to cover other visually observed phenotypes in these and other plant taxa.

To reduce semantic ambiguity and heterogeneity and achieve finer granularity, very specific definitions for ontology terms need to be provided. Though in some cases, devices or gauges are available that can make these specific definitions, most semantic concepts related to visually observed phenotypes are either difficult or tedious to manually quantify (e.g. color, disease resistance) or easy to measure but difficult to partition measurements into semantic terms. For example, plant height is an easy trait to measure; however, partitioning the continuum of heights for a specific kind of plant into terms like "dwarf", "short", "average", "tall" can be more difficult, especially in cases where there are no clear boundaries. How does one decide the quantitative boundary between these semantic terms?

Our approach, which utilizes a training set of phenotype imagery annotated with semantic labels in conjunction with data mining techniques, facilitates both the determination of very specific definitions of these vague semantic concepts and, in doing so, allows the computer to learn the quantitative boundary between concepts within the same semantic class. Imagery was chosen because many of the relevant semantic terms, which may or may not be included in existing bio-ontologies, can be accurately quantified using computer vision and image processing (CV/IP) algorithms. In order to show the utility of computational algorithms for measuring a variety of semantic concepts, we provide below sample measurements for each characteristic discussed to illustrate the capability and necessity of CV/IP algorithms as part of the ontological framework. This work is not intended to introduce novel or improved algorithms for phenotype quantification, but rather to illustrate the power of computational methods for quantifying phenotypic appearances and to use the measurements from these methods to better define semantic concepts.

**Figure 2:**
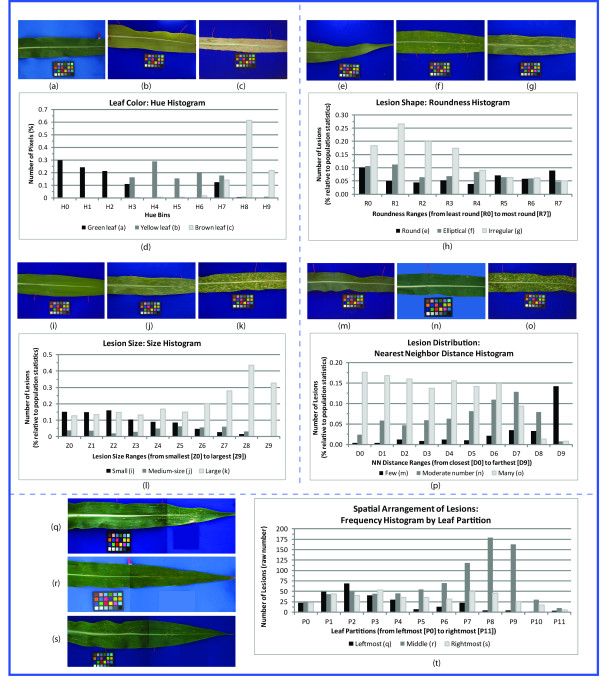
**Example measurements of lesion characteristics in *Zea mays *using imagery and computer algorithms**. Many useful phenotypic attributes can be quantified via calculations from phenotype imagery. Here, we show a variety of phenotype appearances in maize as well as corresponding measurements to demonstrate the capability of algorithms in differentiating these appearances. Each section of the figure contains a series of images that vary by some characteristic as well as a chart showing the differences in the measurements (features) of this characteristic from the images. The top left section contains images of leaves that are (a) green, (b) yellow, and (c) brown. The color differences are clearly visible in the (d) chart of corresponding histograms based on the hue of the leaves. Similarly, the top right section contains leaves with lesions of varying shape: (e) tiny round lesions, (f) elliptical lesions, and (g) irregularly shaped lesions. A (h) histogram of lesion roundness shows how shape can be measured in a way to differentiate these lesion shapes. In the middle left section, leaves with (i) small, (j) medium, and (k) large lesions are shown, along with (l) a histogram of lesion size. The right middle section attempts to capture lesion distribution in leaves with lesions ranging from sparse to dense (m)-(o). An algorithm that plots a (p) histogram of the distance to the nearest neighboring lesion is used to provide a measure of lesion distribution. Finally, the bottom section contains (q)-(s) leaves with lesions in various spatial arrangements. By vertically partitioning a leaf into sections of equal width, the spatial configuration of lesions can be measured by counting the number of lesions in each partition, and this histogram is shown in (t).

Maize is used as the primary prototype for the development and demonstration of this framework. After studying and categorizing descriptions of maize lesion mimic mutants and corn plants afflicted with SLB, the prevailing characteristics in these descriptions were determined. These characteristics include color, size, shape, frequency/distribution, and spatial/anatomic relationships. We discuss the importance of each of these characteristics with respect to our example maize phenotypes below. Figures 2 shows a summary of the various maize phenotypes and some example measurements that can be captured by image processing algorithms.

#### Color

Color is very important in the characterization of many plant traits (e.g. leaf color, stem color, seed coat color, fruit color), the analysis of several diseases, as well as the description of mutants. For maize lesion mimic mutants (*les *mutants), color can be used to determine whether a lesion is necrotic (brown) or chlorotic (green, yellow, or white) and, more importantly, can be used to differentiate mutants, since different mutants produce different colored lesions. Furthermore, color can play an important role in separating normal tissue from abnormal tissue, as evidenced in the SLB scoring annotations. As an illustration, consider the first row of Figure 2, which shows a leaf with a light *SLB *infection, a leaf from one of the *les *mutants, and a leaf with a heavy SLB infection consisting of predominantly green, yellow, and brown tissues, respectively.

Color specification and quantification are both plagued by semantic ambiguity and heterogeneity. Our framework minimizes these semantic issues by precisely describing color using quantitative color profiles rather than semantic terms. For example, consider the different leaves (a, b, c) in the top row of Figure 2. The color variations have been captured computationally using a normalized hue histogram [28,29] (Figure 2 (d)) consisting of 10 equi-width bins. We decided to use the Hue Saturation Value (HSV) color space as it more closely represents the way humans perceive color than the traditional Red Green Blue (RGB) color space. The open-source CV/IP library OpenCV [30,31] was utilized to determine hue values from the images. In this chart, the horizontal axis corresponds to 10 discrete ranges of the hue component, and the vertical axis shows the normalized frequency of each hue range. H0 represents the lowest values of hue, while H9 represents the highest values. The hue histogram shows that the green leaf (a) has significantly higher frequencies in low hue ranges (H0-H3), with a peak in H7 resulting from the brown lesion expression on the leaf. The yellow leaf (b) has values in the middle hue ranges (H3-H7), and the brown leaf (c) in the high hue values (H7-H9). This type of CV/IP algorithm can be used to quantify color in any phenotype.

#### Shape

Shape is an important characteristic that is used to categorize various plant-related structures. This may include the shape of more obvious structures like leaves and fruit, but can also refer to the shape of substructures, e.g. any lesions on the maize leaf surface. The semantics used to describe shape also suffer from semantic vagueness. Consider again the set of maize *les *mutants. The shapes of the lesions in the mutant descriptions in (Figure 2e, f, g) are described as round, elliptical, and irregular, respectively, in [26]. To precisely describe these shapes, one of many shape algorithms can be applied. As a simple example, one could use roundness to help define the shape of a lesion (of maximum diameter *d*), which is defined as the ratio of the area of a lesion to the area of the circle with diameter *d*. The closer the ratio is to one, the closer the shape of the lesion approaches a perfect circle. (Figure 2h) shows the roundness histogram, also partitioned into 10 equi-width bins, for the *les *mutants in the second row. Roundness bins R0-R9 represent different ranges of roundness from low to high. The leaf with mostly small, round shapes (e) has higher values in the high-roundness bins (R7-R9) than the other two leaves. However, this leaf (e) also has some less round lesions, which produce signals in the low-roundness ranges (R0-R6). The leaf with fewer round lesions (f) has significantly lower values in the high-roundness partitions (R8-R9) and higher values in the low-roundness partitions (R1-R2) than leaf (e). The leaf with many irregular shapes (g) has the highest values in R0-R3. These profiles can be used as an aggregate measure of lesion shape on a leaf.

#### Size

The concept of size is well recognized and established in the existing ontologies and is used in various phenotype descriptions. For maize *les *mutants and SLB infections, the sizes of lesions are typically described as being small, medium, or large. For example, a *les1 *mutant is more likely to have large necrotic spots on its leaves, while *les2 *mutants typically have small necrotic spots. Again, computational methods can be used to minimize semantic vagueness by accurately capturing and representing size terms. To quantify a *les *mutant with respect to size, one would like to measure and record the sizes of all the lesions on a leaf and describe the phenotype as a distribution or aggregation of individual lesion sizes. (Figure 2i, j, k) shows, from left to right, maize leaves with small, medium, and large lesions as well as a chart (l) showing the differences in the lesion size for each of these leaves, with Z0 representing the smallest-sized lesions and Z9 the largest-sized lesions. Each leaf shows higher signals in the area of the histogram corresponding to the size of lesions it contains. It should be noted that the visualized differences in the profile of lesion sizes imply boundaries between the concepts in this semantic class using this computational measure.

#### Frequency and Distribution

Current ontologies like TO and PATO contain some terms related to the frequencies and distributions of various characteristics of plant structures. In addition to these, terms related to the frequency of lesion expression (e.g. "very few", "a few", "moderate", or "many") and the distribution of objects (e.g. "uniform", "clustered", "dense", and "sparse") are necessary to fully describe complex visual traits. In [26], mutants are often defined by the frequency or distribution of lesions: "*les5 *plants are like *les2 *but lesions are more frequent", or "*les7 *plants have evenly distributed spots on the leaf blade" [26].

Because of the subjectivity in human perception, these characteristics are particularly difficult to manually measure and are especially suited for quantification via computational approaches. A simple approach to represent frequency and distribution information is a histogram of distances from a lesion to its nearest neighbor (NN). (Figure 2m, n, o) show three *les *mutants with very different frequencies and distributions of lesions, along with the corresponding NN distance histograms (p). Each partition of the histogram represents a range of NN distances with D0 containing the shortest distances and D9 the largest distances. Histograms with high values in the shorter distance bins (o) are said to have dense expression, whereas sparse expression is represented by higher values in the larger distance bins (m). If a leaf has many lesions, the area under the corresponding plot is larger than the plot of a leaf with fewer lesions. Using measurements such as these, distribution characteristics can be more precisely quantified.

#### Spatial Relationship

Spatial relationships can also be important when describing visual phenotypes and can be expressed in two ways: (1) relative to other objects (e.g. objects are "concentric") or (2) relative to a particular plant structure or landmark (e.g. an object is "2.3 cm from the midrib" or "on the third of the leaf closest to the tip"). PATO contains descriptions of spatial terms from the first category, under the accession PATO:0001631; terms from the second category could be obtained by pre-composing [32] terms from the plant structure ontology (PO) and the spatial (BSPO) ontology from the OBO Foundry [33].

To illustrate how algorithms can be used with these types of terms, consider (Figure 2q, r, s), which contains three leaves whose phenotypes are described with spatial relationships: (q) a leaf with moderate expression on the third of the leaf closest to the stem, no expression on the middle third of the leaf, and some expression on the third of the leaf closest to the tip, (r) a leaf that has small lesions throughout the leaf with particularly high expression on the fourth of the leaf closest to the tip, and (s) a leaf with quite uniform expression on the entire length of the leaf. By dividing the leaf into different partitions, we can more precisely quantify some of the different spatial characteristics found in these leaves. For example, (Figure 2t) contains a histogram of the number of lesions in each partition. Note that the image characteristics described above can be clearly seen in this plot.

### The Computable Visually Observed Phenotype Ontological Framework

**Figure 3:**
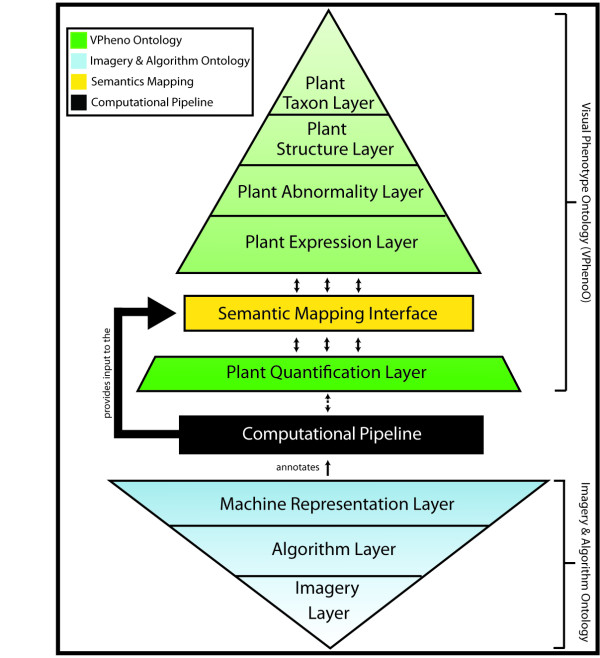
**The four components of the CVOPOF and the relationships between them**. The first component is the VPhenoO (in green), which is the organization of semantic concepts related to phenotype descriptions. This includes everything from species identifiers to qualitative descriptors to quantitative measurements. The quantitative measurements correspond to sets of ranges of measurements, and the linkages between these layers are computed via the semantic mapping interface (in yellow). The imagery and algorithm ontology (in blue) hierarchically relates terms related to images, algorithms, and measurements. The final component, the computational pipeline (in black), defines the imaging protocol and processing plan for phenotype images. It is constructed using terms from the imagery and algorithms ontology, and the measurements it produces provides the input that allows the semantic mapping interface to compute association rules linking measurements to semantic concepts.

In order to formalize the modeling, representation, and quantification of high-level semantic concepts used in phenotype descriptions, the Computable Visually Observed Phenotype Ontology Framework (CVOPOF) for plants has been designed. This framework maintains interoperability with existing bio-ontologies by interfacing species-specific characteristics, anatomical structures, phenotypes, and trait concepts with the bio-ontologies PO, TO, and PATO. Because the qualitative terms of the current bio-ontologies suffer from ambiguity, heterogeneity, and granularity problems, CVOPOF links them to low-level quantitative measurements in a computational way, by evaluating imagery content of visually observed phenotypes. CVOPOF increases the utility of bio-ontologies, especially in terms of information retrieval (IR). As indicated in Figure 3, the framework is divided into four components: a visual phenotype ontology (VPhenoO), an ontology for imagery and computer algorithms, an annotated computational pipeline, and a semantic mapping interface to link the ontologies together.

#### Visual Phenotype Ontology

In this first portion of the CVOPOF framework, we introduce the structure for a new ontology skeleton called VPhenoO. This aspect of the framework is constructed to relate and organize the terms used in phenotype descriptions and annotations. This is accomplished by leveraging well-established bio-ontologies and including additional terms where necessary. A VPhenoO can be divided into the following five layers, which define the domain for the ontology, the first four of which are formed by interfacing to other established ontologies/terminologies:

• The *Plant Taxon *layer is linked to the plant taxonomy classification http://www.ncbi.nlm.nih.gov/Taxonomy/taxonomyhome.html/.

• The *Plant Structure *layer is linked to plant structure identifiers in the Plant Ontology http://www.plantontology.org. This connects to the *Plant Taxon *layer using "part of" relationships.

• The *Plant Abnormality *layer is linked to corresponding species-specific information such as disease, mutation, etc. Maize lesion mimic mutants (MaizeGDB, http://www.maizegdb.org), for example, represent a class of mutations. The ontological relationship used to connect a plant abnormality to the *Plant Structure *layer is an "expressed on" relationship.

• The *Phenotype Expression *layer contains semantic concepts found in phenotype descriptions, many of which can be linked to TO and PATO, which identify quality bearing traits and qualitative values for those traits http://www.gramene.org/plant_ontology/; http://bioontology.org/wiki/index.php/PATO:Main_Page. An "expressed by" relationship is used to join this layer to the *Plant Abnormality *layer.

• The *Phenotype Quantification *layer contains more precise quantitative values for the semantics in the layer above. This layer, which uses a "describes" relationship to connect to the *Phenotype Expression *layer, will be discussed in more detail after the semantic mapping interface.

**Figure 4:**
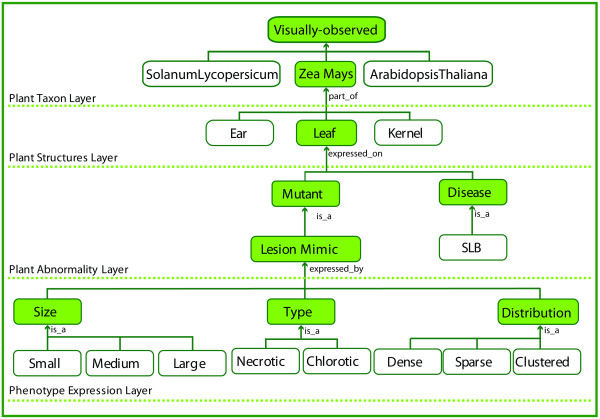
**Hierarchical structure of a partial Visual Phenotype Ontology (VPhenoO)**. A snapshot of the top four layers (separated by dotted lines) of a sample VPhenoO is displayed, showing how semantics related to maize lesion mimic mutants might be organized in the ontology. The top two layers also contain additional species and structures, respectively, demonstrating the entry points where phenotype semantics related to these would be located. The terms highlighted in green indicate terms that have been expanded.

In Figure 4, the conceptual schema for the first four layers of a sample VPhenoO for lesion mimic mutants in maize are shown. The included semantics in this figure are not meant to represent the complete set of relevant terms, but rather just a sample of a VPhenoO's structure.

#### Ontology of Imagery and Computer Algorithms

Since imagery is used in CVOPOF to computationally determine the correspondence between semantic concepts and quantification of these concepts, an ontology to organize the information related to imagery and computer algorithms used to process imagery was included in the framework. The rationale is twofold. First, it provides a listing of the types of measurements that can be made from image content using computer algorithms. Second, by providing this listing, the ontology helps a user group construct an imaging pipeline (see the next section) for standardized imaging and processing of phenotype images. This also ensures that measurements taken from these images are comparable, which is a prerequisite for utilization of our semantic mapping module.

**Figure 5:**
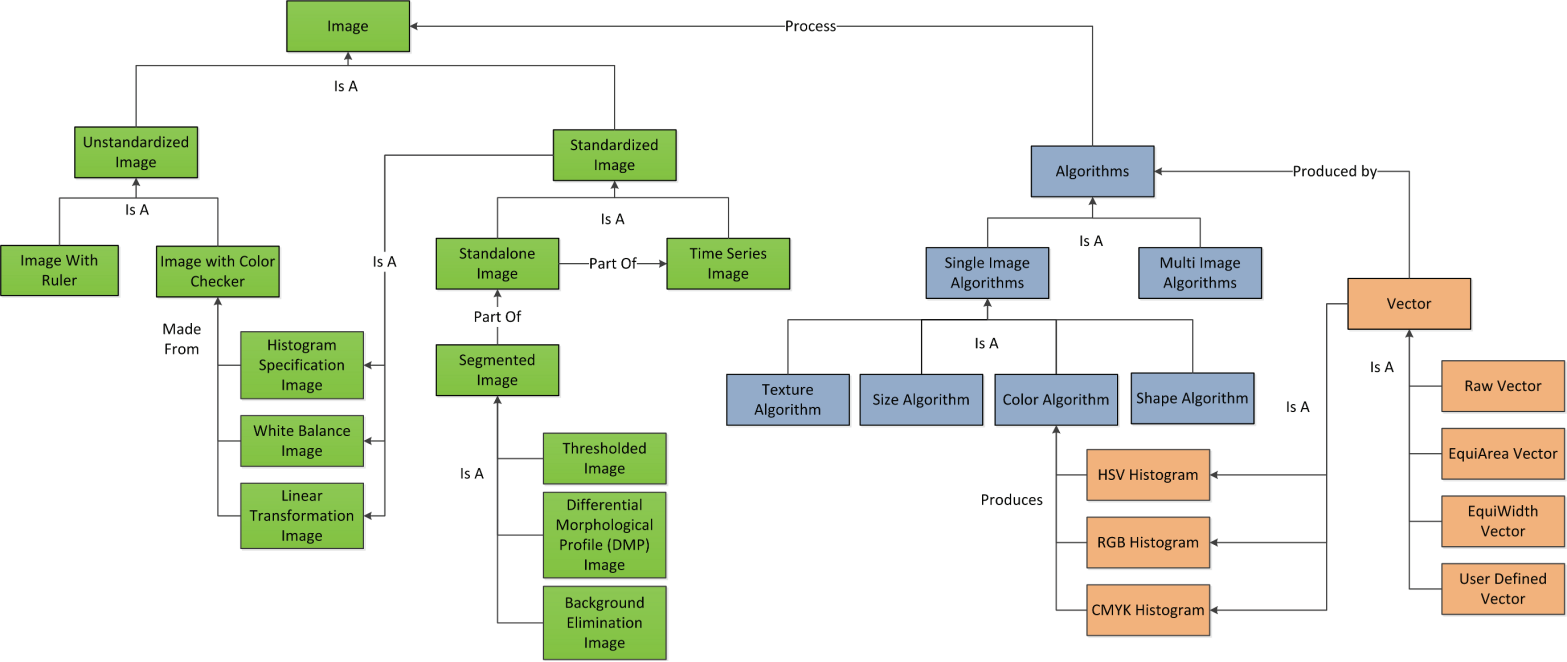
**Sample of an imagery and algorithms ontology**. The ontology covers imagery and algorithms through the steps in computational processing including preprocessing, feature extraction (phenotype measurement), and postprocessing (normalization). The imagery portion (left, in green) categorizes imagery transformations during the preprocessing stage. This includes image standardization, segmentation, and grouping. The algorithm portion (middle, in blue) covers the algorithms involved in feature extraction. Each of these algorithms produces vectors, or measurements, as output. The algorithms are categorized by the number of input images and by the trait being measured. Finally, the vector portion (right, in orange) of the ontology handles postprocessing of the measurement vectors.

The semantics in an ontology of imagery and algorithms can be divided into three layers, as depicted in Figure 3. The *Imagery *layer is the top layer (see the bottom of Figure 3) and contains semantics related to imagery. Images may be categorized by their adherence to certain imaging protocols. These protocols facilitate the use of certain normalization algorithms to transform them to baselines where the images become comparable. An imaging protocol may correspond to the inclusion of a color or size standard placed in the field of view or the use of a certain background type or color when imaging a phenotype. Images may also be classified by whether they exist on their own or as part of a series of images to capture a temporal phenotype. Semantics also exist to describe image transformations that occur during image preprocessing, which may include segmentation or isolation of important aspects of the image. The middle layer in this ontology, the *Algorithms *layer, contains algorithms that produce measurements (features) from imagery. As discussed previously, these features are the low-level quantitative components that are crucial for resolving semantic heterogeneity, facilitating more advanced phenotype searches, and future development of automatic annotation utilities. The algorithms in this layer are classified by the type of information they extract and also by the number of images that are used to produce the information. Finally, the lowest layer is the *Machine Representation *layer, which describes any post-processing transformations done on the output of the algorithms from the layer above, specifically normalization. An example ontology corresponding to the images and algorithms used during the processing of the maize leaf images is shown in Figure 5.

It should be noted that the values of any input parameters for these algorithms are not included in the ontology, as that would add unnecessary complications. Since the values of these parameters determine the quality of the output features, the appropriate parameter values will need to be determined and annotated to a computational pipeline.

#### Computational Pipeline

The consistent and accurate quantification of classes of phenotypes from imagery necessitates the construction of a standard computational pipeline that determines an imaging standard for capture of these phenotypes as well as the set of algorithms used to produce the required features for representing specific phenotypic traits. The benefit of such a pipeline is that all phenotype images adhering to the standard and processed using the pipeline will be comparable in terms of high-level semantic descriptions as well as low-level computational features. This is an essential aspect of the semantic mapping procedure discussed in the next section, as images are not computationally comparable if (1) they cannot be transformed to a common baseline, i.e. if they do not adhere to some imaging standard, or (2) there is a not a common set of features collected for the entire set. The pipeline will guide image processing from an un-standardized, raw image through pre-processing, feature extraction, and post-processing (which includes feature normalization), all of which are covered components of an ontology of imagery and algorithms. The pipeline can be annotated with the described ontology and extended so that the appropriate input parameters for each algorithm are stored.

**Figure 6:**
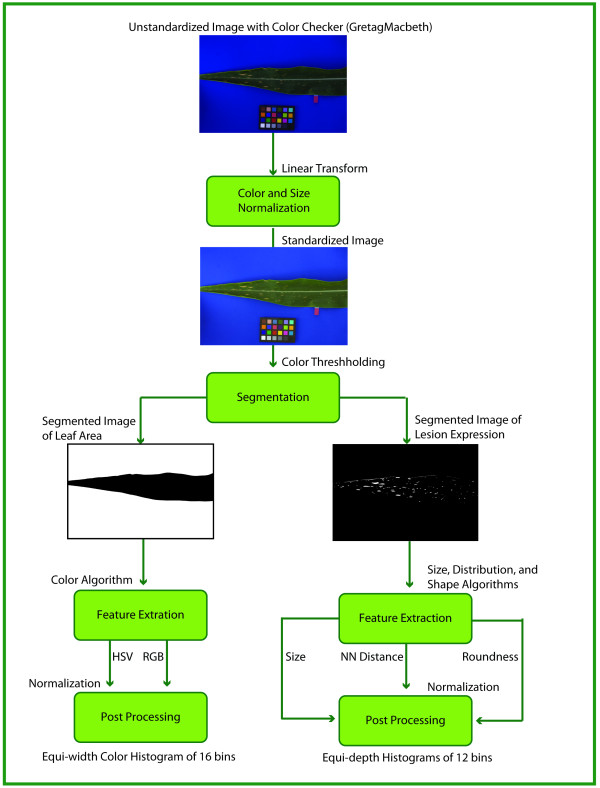
**Sample computational pipeline for the maize leaf imagery**. This flow chart illustrates the entire process from raw phenotype image to phenotype measurement extraction. The input image indicates the required imaging protocol for use of this pipeline, namely the use of a color checker and a standard solid background color. The inputs of the pipeline (through feature extraction) are all images, with the boxes corresponding to algorithms applied to the input images. The postprocessing boxes indicate transformations of the raw measurements to their final form. It should be noted that each step is annotated with terms from the imagery and algorithms ontology and augmented with additional necessary parameters. This pipeline provides a standard processing path for phenotype imagery and also provides the necessary input for the semantic mapping module.

As a concrete example, consider the annotated computational pipeline in Figure 6 constructed for capturing and processing maize leaf phenotypes. The input and output to each step in the processing pipeline correspond to a value in the imagery and algorithms ontology, and these terms have been supplemented with parameter information to provide additional algorithmic detail.

#### Semantic Mapping

The most critical part of the framework is the semantic mapping interface, which is the computational module that utilizes a data mining approach to automatically learn the quantitative boundaries between semantic concepts. Input to this module consists of a set of phenotype images that have been (1) processed using the computational pipeline to measure the desired phenotypic characteristics (i.e. image features extracted) and (2) manually annotated (by one or more curators) with the semantic concepts of interest. For example, Table 1 shows selected features extracted for the Anna Russian tomato variety (labeled by SGN as medium in size and oxheart in shape) using Tomato Analyzer. Once all this information for all the tomato fruits for which we have measurements and semantic labels has been amassed, it is passed to this module for processing.

**Table 1:** Example of extracted features for the Anna Russian tomato variety.

Num	Perimeter	Area	Max Width	Max Height	Curved Height	Shape Index 1	Shape Index 2	Curved Shape Index
01	787.2	41884	228	237	252.3	1.04	0.98	1.12

02	813.1	43809	230	245	268.6	1.07	1.04	1.22

03	719.4	34326	192	237	261.4	1.23	1.23	1.37

04	719.4	34807	189	238	262.9	1.26	1.27	1.42

05	762.4	39612	218	248	276.9	1.14	1.14	1.28

06	793.6	41503	221	256	286.0	1.16	1.15	1.33

07	753.1	37440	221	224	266.4	1.01	0.98	1.22

08	746.7	38172	220	220	263.9	1.00	0.99	1.23

This tomato variety is labeled by SGN as being "medium" in size with an "oxheart" shape. The features were extracted using the Tomato Analyzer software.

Our semantic mapping module is a knowledge discovery process that determines complex and flexible association rules among a sufficiently large training set of visual semantics and machine-readable features from plant images. Our approach emphasizes the use of flexible semantics to address the heterogeneous semantic assignments that are inherent in the descriptions of plant phenotypes. A key to the success of the predictive power of our algorithm is to identify a training dataset that would return accurate models while being minimal in size to reduce the burden on those imaging and manually annotating the phenotypic appearances. While some approaches suggest large amounts of training data [34] to compensate for poor training data choice, other research [35] shows that prediction gain decreases with adding more data to the training and eventually reaches a plateau where adding more training data does not bring any improvements in performance. In our approach we capitalize on the tradeoff between size and quality of training data. We choose the training data to contain representative examples from all semantic assignments.

We model the mapping between semantic and machine-readable features by creating a set of association rules [36,37] via data mining. The association rules in our model are mined using the Total-From-Partial tree structure [38] over the entire image database with semantic assignment for phenotypic expressions. Each association rule *r *(*A*→*C*) has a set of feature value ranges as its antecedent (*A*) and a semantic as its consequent (*C*). An example rule is {*F*006 ∈ [0.09, 0.16] ʌ *F*399 ∈ [0.12, 0.21]}→ *ellipticallesion*. This rule maps the semantic "Elliptical Lesions" into a two-dimensional feature subspace of low-level features F006 and F399 (see Table 2 for feature explanations). A new image is considered relevant to the semantic "elliptical lesion" if its feature measurements fall in the specified range. To make the rules more flexible, after the association rules are discovered, the antecedents are refined through fuzzification; the crisp feature intervals in the antecedents are replaced with possibility distributions that model the relevance of the individual feature subspace. The following equation is the asymmetric possibility function used to model the semantic assignment for a feature subspace ϑ of a feature *f*. The shape of this function is controlled by three parameters: center - *λ^1^*, width - *λ^2^*, and slope - *λ^3^*.

**Table 2:** All features extracted for the maize leaf phenotypes, a total of 452 features.

Features	Feature Class	Relevant Pixels of the Leaf	Number of Features
001 - 048	RGB histogram	Entire leaf	48 (16, 16, 16)

049 - 096	RGB histogram	Necrotic lesions	48 (16, 16, 16)

097 - 144	RGB histogram	Chlorotic lesions	48 (16, 16, 16)

145 - 192	RGB histogram	Non-lesions	48 (16, 16, 16)

193 - 239	HSV histogram	Entire leaf	47 (15, 16, 16)

240 - 286	HSV histogram	Necrotic lesions	47 (15, 16, 16)

287 - 333	HSV histogram	Non-lesions	47 (15, 16, 16)

334 - 380	HSV histogram	Chlorotic lesions	47 (15, 16, 16)

381 - 392	Size histogram	Necrotic lesions	12

393 - 404	Size histogram	Chlorotic lesions	12

405 - 416	Roundness histogram	Necrotic lesions	12

417 - 428	Roundness histogram	Chlorotic lesions	12

429 - 440	NN distance histogram	Necrotic lesions	12

441 - 452	NN distance histogram	Chlorotic lesions	12

All color features (red, green, blue, hue, saturation, and value) were generated using equi-width binning, and all lesion features (size, roundness, nearest neighbor (NN) distance) were generated using equi-area binning. The numbers in parenthesis in the Number of Features column show the total number of features as well as the number per channel for the RGB and HSV histograms.

**Figure 7:**
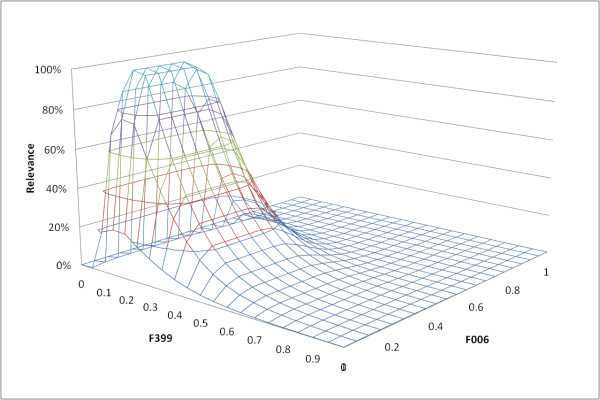
**Visualization of an example fuzzified association rule that maps the semantic "elliptical lesion" into a 2D feature space**. A crisp association rule generated from data mining has been transformed so that images relevant to this semantic can be measured more continuously, instead of in a binary fashion with the crisp rule.

Figure 7 shows the sigmoid mapping of the semantic "elliptical lesion" into a two-dimensional space formed by the low-level features F006 and F399. According to this chart, an image having feature values between 0.107 and 0.196 for F399 as well as between 0.08 and 0.14 for F006 has a high probability of being relevant to the "elliptical lesion" semantic. When feature measurements are further away from this interval, the relevance of an image to this semantic decreases. For example, an image with feature values of 0.96 for F399 and 0.94 for F006 has a very low probability to be relevant to "elliptical lesion". The association rules are then indexed and linked to the proper nodes in the ontology. For more details regarding the construction of such a semantic map, the reader is referred to [39]. It should be noted that the accuracy of this semantic mapping, and hence the accuracy of any applications utilizing this framework, will rely heavily on a sufficiently number of phenotype images/descriptions annotated using the framework.

**Figure 8:**
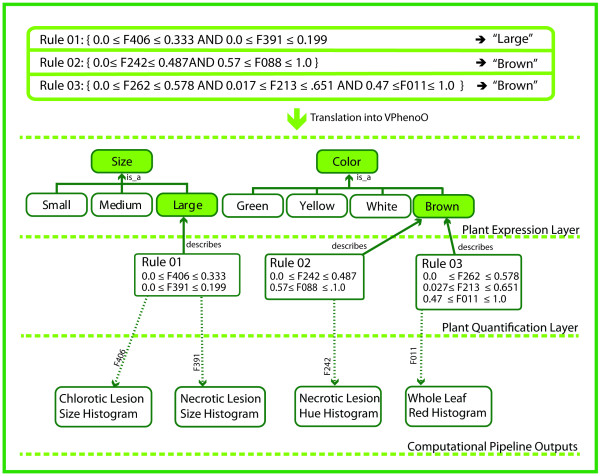
**Embedding association rules from semantic mapping into a VPhenoO**. Three example association rules obtained from semantic mapping are shown in the table above. Each rule contains a region of feature space (sets of ranges of phenotype measurements) as the antecedent and a semantic concept from the *Plant Expression *layer as the consequent. These rules become the *Plant Quantification *layer in a VPhenoO and are linked by the consequent to the layer above. There are also implicit links between the *Plant Quantification *layer in a VPhenoO and the computational pipeline, as the measurements in each antecedent are linked to specific vectors obtained using specific algorithms from specific images.

The rules from the semantic mapping module become the concepts in the fifth and final layer of a VPhenoO, the *Plant Quantification *layer. Figure 8 shows three rules that were derived from the semantic mapping module for our maize leaf image collection (full results on this collection are given in the next section) in addition to the encoding of these rules into the sample VPhenoO. The linkage of the semantic categories to these rules provides the concrete tie between the measurements extracted from the phenotype image content and the meaning of the semantic concepts. Representing this layer in this way has the added benefit of providing implicit linkages between the *Plant Quantification *layer and the computational pipeline, as each feature represented in a rule's antecedent is linked to the algorithm that generated that feature.

## Results

### Maize Lesion Mimic Mutant Phenotypes

**Figure 9:**
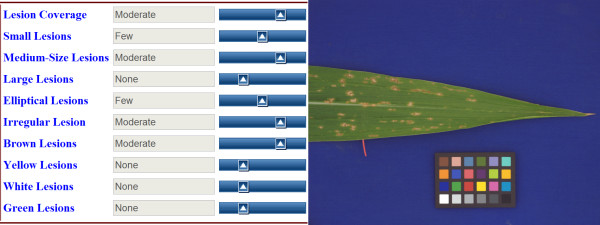
**Example of the set of semantic labels assigned to a maize leaf image**. The curator was asked to assign a degree of relevance to each semantic concept. This figure shows a screenshot of our web-based semantic labeling tool that uses slider bars to simplify the labeling.

The framework was first applied to leaf phenotypes in *Zea mays*. The dataset consists of 310 leaf images covering 15 different lesion mimic mutants taken from a genetics maize field at the University of Missouri. Three different curators assigned semantic labels to each image, and consensus was used to determine the final labels. Semantic categories assigned to the images included terms related to lesion coverage, lesion size ("small", "medium", "large"), lesion shape ("elliptical", "irregular"), and lesion color ("brown", "yellow"). Each semantic label assigned to each image was coded by the curator in terms of degree of appearance, as either "none", "few", "moderate", or "extensive." As an example, Figure 9 shows the consensus labeling for one of the 310 images.

In addition to semantic labeling, each of the maize leaf images was also processed using the computational pipeline in Figure 6. This pipeline consists of a series of C++ computer programs developed in-house for measuring various characteristics of lesion mimic mutant phenotypes. These programs utilize function calls from the open source CV/IP library OpenCV. As a result of this pipeline, a total of 452 features was obtained for each image. Table 2 describes the entire set of features.

The semantic labels and image features were passed to the semantic mapping module. To avoid problems stemming from the curse of dimensionality, this module automatically performs feature selection (genetic, best first, exhaustive search, or greedy stepwise) to statistically determine which measurements are best able to distinguish individual semantics; those features that are not helpful or that are highly correlated with other useful features are excluded before mapping begins. Semantic mapping was performed individually on each class of semantics (e.g. lesion size). Multiple rules were generated for each semantic term, and a summary of the outputted rules are shown in Table 3. The full set of generated rules from this dataset can be found in Additional File 1.

**Table 3:** Summary of the number of mined rules for each semantic concept for the maize leaf phenotypes.

Semantic Class	Semantic	# of Rules
Lesion Color	Brown Lesions	13

Lesion Color	Yellow Lesions	14

Lesion Shape	Elliptical Lesions	5

Lesion Shape	Irregular Lesions	11

Lesion Coverage	Extensive	17

Lesion Coverage	Moderate	19

Lesion Coverage	Few	12

Lesion Size	Large Lesions	17

Lesion Size	Medium-Size Lesions	3

Lesion Size	Small Lesions	10

**Figure 10:**
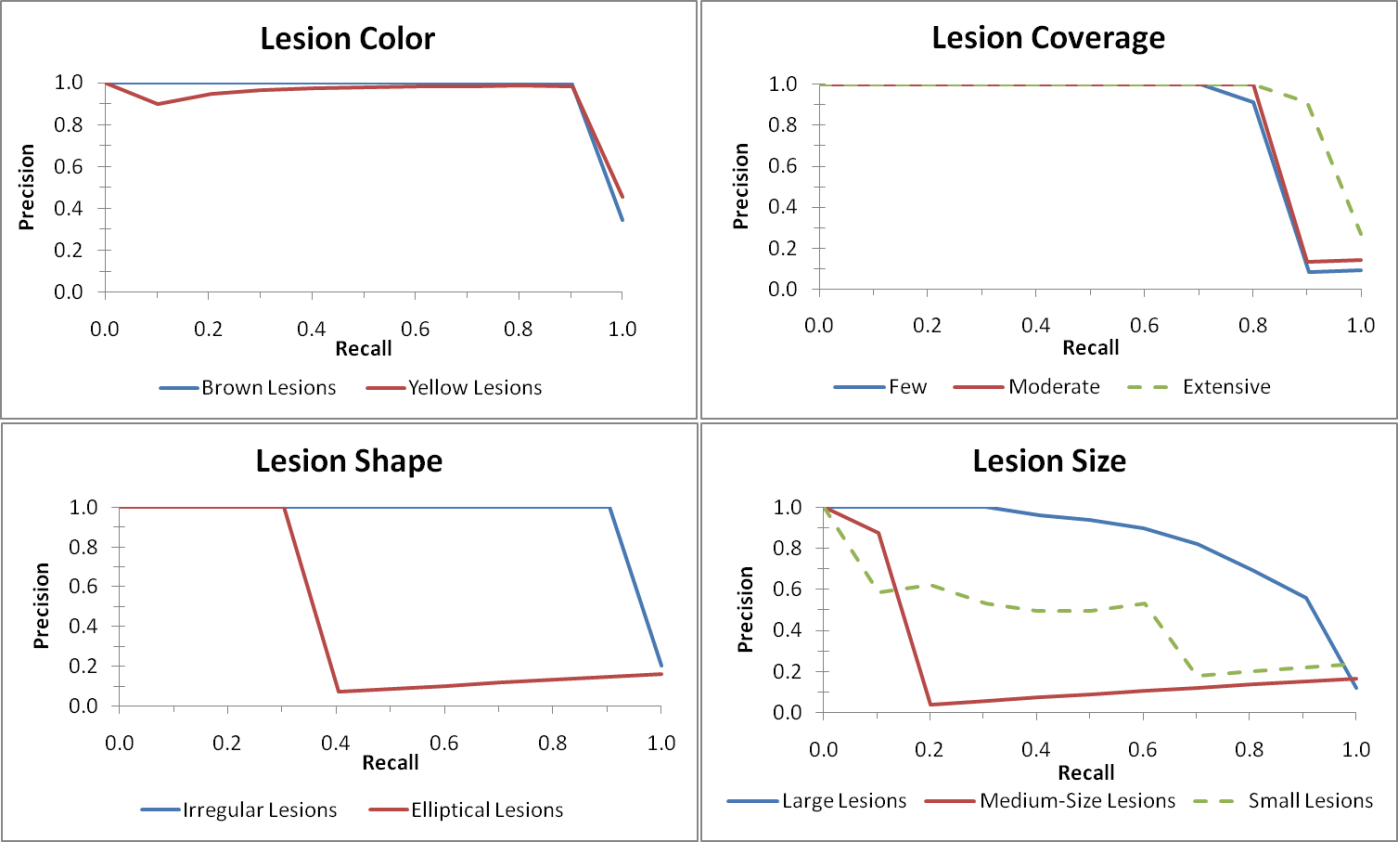
**Precision-recall curves for the mined results for the maize leaf phenotypes**. The curves are separated by semantic class: lesion color (top left), lesion size (top right), lesion shape (bottom left), and lesion coverage (bottom right).

**Figure 11:**
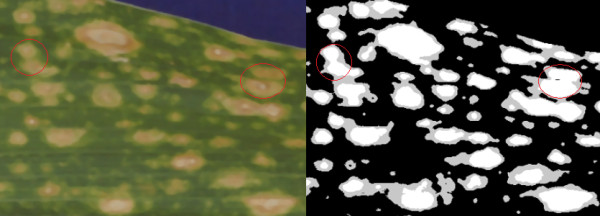
**Example maize leaf image along with an image showing the segmented lesions**. Two examples are shown (in the red circles) where necrotic lesions appear to be separate by human eye, but the lesion segmentation algorithm merges them into a single lesion since the areas of necrosis are actually touching. White pixels in the segmented image correspond to areas of necrosis, and gray pixels to areas of chlorosis.

We also conducted experiments on the generated rules to evaluate how well the rules were able to predict the chosen semantic labels. To determine the quality of the generated rules, a resubstitution experiment was carried out. The mean average precision (MAP) for each of the semantic categories as well as the average MAP for each semantic class are shown in Table 4. Precision-recall curves were also generated (see Figure 10). Inspection of these results shows highly accurate results for lesion coverage and lesion color with decreasing quality of results for lesion shape and lesion size. The reason for the decline of results in these latter two semantic classes becomes obvious when one looks more closely at lesion segmentation in many of these images. Though human perception is able to project boundaries between individual lesions in images like Figure 11, upon closer inspection of the image (and evidenced by the segmentation algorithm) many of these so-called individual lesions are in fact touching and should be treated as a "single" larger lesion. While this visual illusion will not have an effect on the lesion coverage or color semantics, it will definitely have an effect on lesion sizes and shapes.

**Table 4:** Mean average precision (MAP) of maize leaf semantic assignment.

Color Semantics	MAP	Shape Semantics	MAP
Brown Lesions	1.000	Irregular Lesions	1.000

Yellow Lesions	0.962	Elliptical Lesions	0.727

*Color Average*	*0.981*	*Shape Average*	*0.864*

**Size Semantics**	**MAP**	**Coverage Semantics**	**MAP**

Large Lesions	0.970	Few	0.986

Medium-Size Lesions	0.432	Moderate	0.993

Small Lesions	0.582	Extensive	0.998

*Size Average*	*0.661*	*Coverage Average*	*0.992*

### Tomato Fruit Phenotypes

In addition to *Zea mays*, the framework was applied to some fruit phenotypes in a second species, *Solanum lycoperiscum*. This dataset consisted of 20 tomato fruit images, each of which contained 2 to 16 individual fruits, from 19 different tomato varieties. Table 5 provides the number of fruits from each tomato variety. These images in addition to the semantic labels associated with the different tomato varieties were obtained from SGN. The semantic labels indicated the shape and the size of the tomato fruit.

**Table 5:** Number of tomatoes for each variety.

Name	# of Fruits	Name	# of Fruits
Anna Russian	8	Galatino	16

Banana Leg	8	Goliath	10

Belmonte	6	Grape	16

Black Plum	16	Green Grape	8

Borgo Cellano	12	Grushovka	2

Corbarino	12	Guajito	8

Cuban Yellow Grape	16	LA1312	12

Determinato Tondino	8	LA2294	12

Druzba	8	LYC1891	6

Fiascetto	10		

In this case study, instead of developing a brand new set of algorithms, as was required for the maize case study, an available tool from the plant community, the Tomato Analyzer [6,40], was used for processing the images. A total of 56 features was extracted from each tomato fruit using this software, and Table 6 provides a listing of those features. The semantic module was again applied to these semantic labels and image features. A summary of the generated rules is provided for this dataset in Table 7, and the complete details about all the mined rules can be found in Additional File 1.

**Table 6:** Features selected and extracted for tomato fruit phenotypes from Tomato Analyzer.

Features	Description	# of Features
1	Fruit Perimeter	1

2	Fruit Area	1

3	Width at Middle of Height of Fruit	1

4	Maximum Width of Fruit	1

5	Height at Middle of Width of Fruit	1

6	Maximum Height of Fruit	1

7	Curved Height of Fruit	1

8-10	Shape Index of Fruit	3

11-13	Average RGB values	3

14	Average Luminosity	1

15-17	Average L*a*b* values	3

18	Average Hue	1

19	Average Chromatic	1

20-56	HCL histogram	37

**Table 7:** Summary of number of mined rules for each semantic concept for the tomato fruit phenotypes.

Semantic Class	Semantics	# of Rules
Fruit Shape	Flat shape	8

Fruit Shape	Heart shape	5

Fruit Shape	Long shape	2

Fruit Shape	Obovoid shape	1

Fruit Shape	Oxheart shape	2

Fruit Shape	Rectangular shape	1

Fruit Shape	Round shape	1

Fruit Shape	Ellipsoid shape	13

Fruit Size	Small size	5

Fruit Size	Medium size	8

Fruit Size	Large size	4

**Figure 12:**
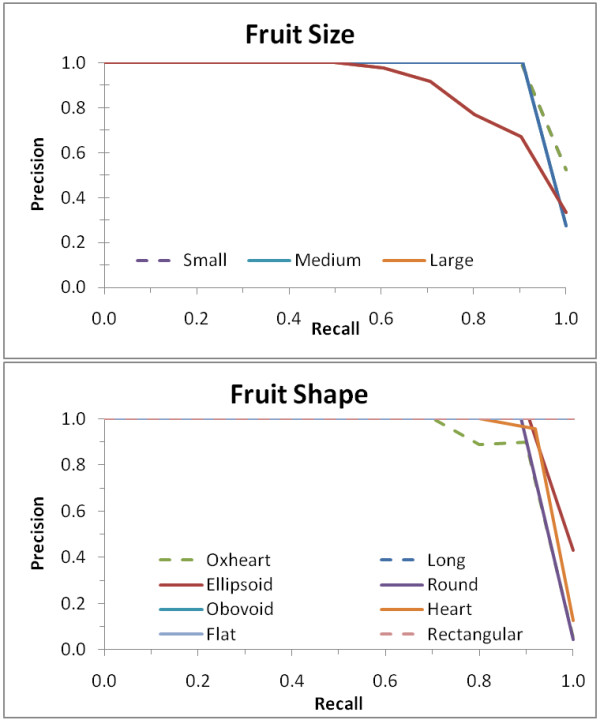
**Precision-recall curves for the mined results for the tomato fruit phenotypes**. The curves are separated by semantic class: fruit size (top) and fruit shape (bottom).

The same resubstitution experiment was conducted on this dataset to once again verify the quality of the image features and generated rules. The MAP values (see Table 8) and precision-recall plots (see Figure 12) again show high quality results for all semantic categories. It is noteworthy to compare the MAP results between maize and tomato. The rules for the tomato fruit have higher precision results than those rules for maize leaves. This is because, in the current collection, the maize leaf phenotype expression is more complicated, in terms of appearance, measurement, and semantic labeling, than the tomato fruit phenotype expression. This is evidenced by the increased number of rules linked in the ontology for maize leaves.

**Table 8:** Mean average precision (MAP) for tomato fruit semantic assignment.

Shape Semantics	MAP	Size Semantics	MAP
Oxheart shape	0.985	Small size	1.000

Long shape	1.000	Medium size	0.999

Ellipsoid shape	1.000	Large size	0.998

Round shape	0.988	*Size Average*	*0.999*

Obovoid shape	1.000		
		
Heart shape	0.998		
		
Flat shape	1.000		
		
Rectangular shape	1.000		
		
*Shape Average*	*0.996*		

### Merged VPheno Ontology for Multiple Species

A VPheno Ontology containing semantics from maize leaf lesion phenotypes and tomato fruit phenotypes was constructed from the two case studies presented above. This ontology contains semantics in all five of the described layers in a VPhenoO, from taxonomic classification to semantics describing phenotypic appearance to automatically determined association rules linking semantic concepts to measurements of these phenotypes from imagery. The maize portion of the ontology contains 162 nodes - 40 of which are high-level semantics with 122 corresponding to the generated semantic rules for the maize leaf. The remaining part of the ontology is related to the tomato fruit phenotypes and consists of 68 nodes - 19 high-level semantics and 49 generated rules. This ontology is available, in Web Ontology Language (OWL) format, in Additional File 2.

These case studies explicitly demonstrate how CVOPOF utilizes a variety of established ontologies including PO, TO, and PATO, and also the framework's flexibility in being able to accommodate both customized algorithms as well as publically available computational tools (e.g. Tomato Analyzer).

## Discussion

### Applications

**Figure 13:**
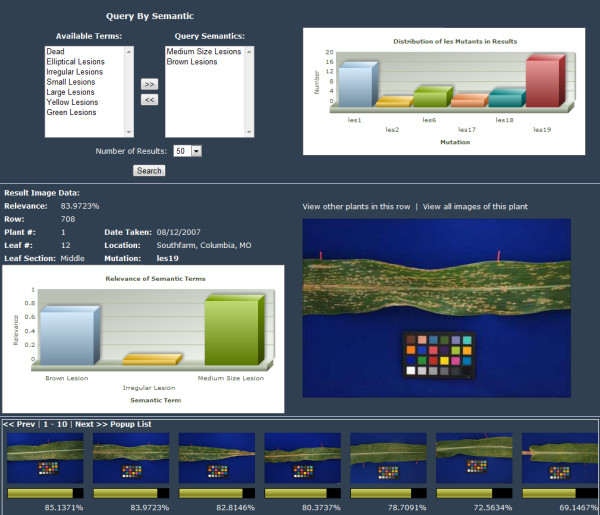
**Screenshot of an example semantic search result page**. Using the CVOPOF framework, advanced search mechanisms, like the semantic search above, can be constructed. In VPhenoDBS http://phenomicsworld.org, the semantic search feature allows a user to search for relevant phenotype images by querying with a list of semantic terms; in this example, the user is searching for "Medium Lesions" and "Brown Lesions." The images searched are not physically tagged with semantic labels; rather, the framework allows the semantics (via the semantic mapping module) to be converted and searched in the phenotype feature (measurement) space. The result page shows (bottom pane) the top ranked results; (middle pane) information about the currently selected result image including the image itself, any textual metadata, and all semantics with non-zero relevance extracted for this image; and (top right) the distribution of *les *mutants in the top ranked results.

The semantic mapping module provides the groundwork for two advanced phenotype applications: retrieval and annotation. Combining a VPhenoO with content-based image retrieval (CBIR) makes efficient querying of visually similar phenotypes from semantics tractable [41,42]. Semantic searches use canonical semantics and/or phenotype annotations as queries. An example system, illustrated in Figure 13, shows a semantic search for maize lesion mimics. In this example, a user selects a combination of two semantic terms ("*Medium Size Lesions*" and "*Brown Lesions*") to search for relevant phenotype images from the database. For each semantic term, a list of the most relevant images is formed. The image lists are fused together to create a ranked list of images that best match all semantics and are returned for the user to consult.

In addition to semantic search, the semantic mapping module also facilitates an annotation utility. With this application, a user could submit an image to the system, which would generate a feature vector using the devised computational pipeline. Feature values would be matched to antecedents to find applicable association rules, and then through probabilistic means, those rules would be used determine the relevance of each semantic to the image. This is also part of the application shown in Figure 13. For the result image shown in the middle pane, the list of semantics with non-zero relevance to this image is displayed in the bar chart to the left of the image. The height of each bar corresponds to the relevance of the corresponding semantic.

### Potential Applications

Use of the CVOPOF framework makes possible the development of a number of more advanced applications and utilities for analysis and annotation. First, when images have corresponding descriptions annotated using a VPhenoO, CBIR searches could be used to facilitate studies of the similarities and differences in semantic terms used to describe visually similar phenotypes, perhaps across anatomical structure and even taxa. In addition, one could consider using the semantic mapping module to generate association rules for each of several individual curators. With the generated rules, one could quantify and perform statistical analyses on the similarities and differences between individual curators' perceptions. Once the system learned the annotation style for an individual, the system could then allow database curators to detect inconsistencies or possible errors in annotation so as to maintain high levels of repository integrity. Alternatively, the rules from individual curators could be combined to form "consensus" rules, which could represent a standard for annotation of particular phenotypes.

The framework could also be used for training purposes. A trainee could use the system to become familiar with the semantics of phenotype descriptions by examining annotated descriptions in conjunction with phenotype imagery. The training should improve the consistency of newly assigned phenotype descriptions as well as human perception regarding plant phenotypes.

### Utilization of the Framework

In order to fully benefit from this framework, a great deal of collaboration, commitment, and careful planning by the plant community are required. This includes everything from construction of the ontologies and labeling of image examples by a central administrative group to utilization of the unified information system by more peripheral end users in that plant phenotype's domain.

To start the process, an administrative group must be formed. This group should contain experts from the particular taxon/phenotype community as well as CV/IP experts. This collaboration will be vital to the success of the framework, as both groups bring expertise that is essential for correctly identifying and measuring phenotype expression. The administrative group will first identify those phenotypes of interest that can be captured and represented through imagery. The distinguishing characteristics of these phenotypes must then be determined, and, with the aid of computer vision experts, the algorithms and parameters necessary to quantify those traits need to be selected.

Once these items are determined, attention can be turned to ontology creation. The top layers in the VPhenoO can be constructed with either semantic terms present in existing ontologies or by new terms. Though ontology construction will be tedious, the realized benefit of minimizing semantic vagueness in the descriptions should be worth the effort.

In parallel with ontology construction, a pipeline for processing the phenotype images can be built. This can be constructed and annotated using terms from an ontology of imagery and algorithms. The pipeline will ensure that appropriate phenotype measurements will be made for each image submitted to the system. It may also ensure that an image adheres to the defined imaging protocol.

Following these steps, a set of training image examples will need to be constructed and annotated. Multiple example images representing each of the various concepts in the VPheno Ontology should be collected and annotated. These training images should also be processed using the pipeline to obtain phenotype measurements from the image content itself.

After these training data are compiled, the semantic mapping module can be executed to determine the correspondences between high-level semantics (ontology concepts) and low-level features (measurements made from phenotype image content). The last step in the initial construction phase of the system is to add the generated rules to the VPheno Ontology.

After the administrative group has created a VPhenoO and computational pipeline and after the semantic mapping module has been trained with the training data, users can begin to take full advantage of the ontological framework. They may submit un-annotated images of their phenotypes to the system. Phenotype measurements will be obtained from the pipeline appropriate to the type of image submitted, and these will be used to automatically generate a list of semantic terms relevant to the image. The list of terms can be reviewed by curators and edited appropriately.

The initial construction phase is only the beginning, as the system is intended to evolve. A number of events will likely occur that the system must be able to accommodate. For example, what will be the process when users request more semantic terms to be added to the ontology? How does the system handle additional training data provided by users who collect, manually annotate, and wish to submit additional phenotype data to the system? If there is additional data for training the semantic mapping module, when is retraining performed? Computer scientists will undoubtedly continue to develop algorithms for measuring various phenotypic characteristics or they may make improvements to existing algorithms. How will the system accommodate inclusion of these algorithms into the processing pipelines?

**Figure 14:**
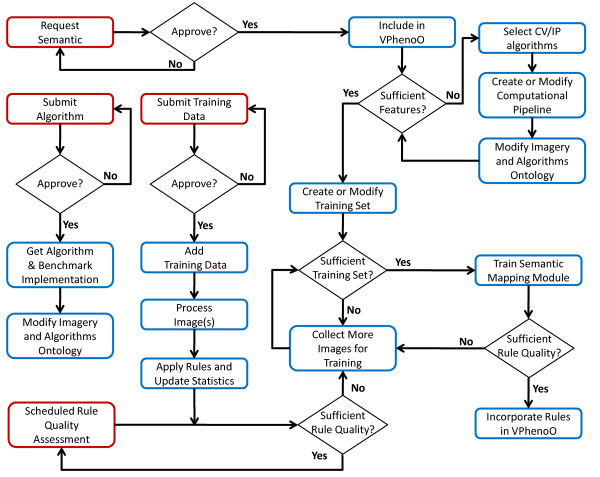
**Flowchart for building the CVOPOF framework**. An instance of the CVOPOF framework represents an evolving system that must be able to accommodate the inclusion of new semantic concepts, algorithms, and training data. The work flow for each of these processes is demonstrated above. Starting points are shown in red.

Figure 14 illustrates the general tasks and processes involved in handling all of these situations. The starting point for each process appears in red. Submission of a new algorithm to the system is a rather simple process. After approval, the administrative group should obtain an implementation of the algorithm and evaluate its performance to verify its ability and accuracy in measuring the claimed phenotype characteristic. Once this has been done, the algorithm can simply be added to the ontology of imagery and algorithms.

The most complicated task is the addition of a new semantic concept. After a request for a new semantic concept has been approved by the central administrative group, the first step will be to include that semantic concept into the VPhenoO. This may simply be adding a related semantic in an existing part of the ontology or it may require an extensive addition to the ontology (adding a new species, plant structure, etc). After this step, the administrative group will need to evaluate whether the semantic can be measured from images in the training data and using algorithms in the pipeline. If the answer is no to either question, the group will need to define the imaging protocol, select CV/IP algorithms, create (or modify) the existing computational pipeline, and make any necessary modifications to the ontology of imagery and algorithms. Once features are decided upon, a sufficient training set will need to be constructed. It is quite difficult to provide specifics on the exact size of a sufficient training set, as it is dependent on several factors including the quality of the image labels and the ease with which the semantic labels in the class can be separated quantitatively. With a good training set, semantic mapping can be performed. The MAP scores and precision-recall curves can be examined to determine the quality of the outputted rules. If the rules show unsatisfactory performance, adjustments to the training set (in terms of size or labels) can be made. If, on the other hand, the rules are of adequate quality, they can be added to the VPheno Ontology in the Plant Quantification layer.

Users may also submit new training data to the system. Approval should be received on the quality of the submitted images and the manual annotations. Afterwards, the image can be added to the training set database and processed by the appropriate pipeline. Using the features extracted from the image, the current set of semantic rules can be applied to the image, which will generate a list of relevant semantic terms. These terms can be compared to the manual annotations, and statistics maintained on the quality of the current rule set can be updated based on this assessment.

Retraining of the semantic mapping module can be initiated in one of two ways. First, if the updated statistics from the added data cause the rule quality to fall below a specified threshold, this could be used to initiate a retraining of the system. Alternatively, an assessment of rule quality could be performed on a regular basis. If the size of the new data is large enough, retraining of the associated rules could be performed. After retraining is accomplished, the updated rules could be included in the VPheno Ontology. The rules that are being replaced could be deleted, deprecated, or made obsolete. Deprecating the old rules would have the benefit of allowing an analysis of the changes in the rules.

While the most extensive and comprehensive benefits from the framework are achievable through widespread collaboration by plant communities, adoption of the framework by smaller cohorts of researchers or even individual research groups can still provide the noted advantages, though on a smaller scale. In this case, the interoperability of phenotypic information modeled by the framework would be limited to only those groups utilizing the framework, which would notably include utilization of the same imaging protocol for phenotype capture. It is noteworthy to mention, however, that any automatic annotations generated through the use of the framework, though not directly comparable, could still be of use to the rest of the plant community.

### Framework Limitations

Though there are many benefits for using the CVOPOF for organization, quantification, and annotation of plant phenotypes, the framework is not without limitations. First, the framework requires a means to make direct or indirect measurements of relevant phenotype characteristics, specifically through phenotype images (though adoption of measurements from other sources would be straightforward). The phenotype images must follow a defined imaging protocol so as to facilitate processing by computer algorithms, and computer algorithms need to exist that can measure the semantics of interest. Second, the semantic mapping module requires sufficient training examples for each semantic concept it is to be trained on. Though it is difficult to specify precisely how many training images are needed per semantic concept, the general rule of thumb is the more training examples the better. Too few training examples can result in no outputted rules from the semantic mapping module, or at the very least rules of poor quality. The MAP scores and precision-recall curves reported above provide evidence of the quality of the generated rules. Implicit in a sufficiently large training set of images is the requirement for semantic labels for each of the submitted phenotype images. This could be problematic as semantic labeling can be a very laborious task. It can also be difficult for humans to label images in an objective and consistent manner. Finally, a potentially major limitation of the framework is that optimal use of the framework will require extensive time, effort, and resources from the plant community to decide upon the species and phenotypes to image, to obtain high-quality phenotype images, to find or develop robust algorithms for measuring phenotypic appearances, to provide consistent and objective labels, and to train and retrain the system as more training data, computer algorithms, and semantic concepts are included. There will also need to be an understanding that this system's accuracy will evolve over time. Despite these limitations, high throughput phenotyping, analysis, and annotation are expected to be critical to ensure rigorous scientific discovery in plant genomic research.

## Conclusions

We have proposed a new ontological framework for visual phenotype semantics that leverages several existing ontologies (e.g. PO, TO, PATO) and expands them, through phenotype imagery and computational processing, to include a robust low-level computational level that facilitates the linkages of high-level semantic concepts found in qualitative observations to quantitative measurements of these concepts from phenotype images. The computational processing aspect of the framework is highly flexible in that it can utilize measurements from existing publically available computational tools (when such tools exist) or from customized algorithms (when public tools do not exist). The linkages are computed by a semantic mapping module that utilizes data mining techniques. The by-product of this framework is the ability to more precisely define phenotypic conceptual semantics in individual plant domains, which leads to a reduction in semantic ambiguity and heterogeneity and improvement in semantic granularity.

This framework facilitates the development of a number of phenotype-related applications. Next generation information retrieval tools like semantic search in addition to automatic semantic extraction from phenotype images have already been demonstrated. With integration of multiple plant structures and species, the framework also has the potential to facilitate phenotype retrievals across plant structure and species, though more investigation is required. In addition, advanced and comprehensive phenotype annotation analysis could be performed by applying various data mining and knowledge discovery tools to a repository of images annotated using the framework.

## Competing interests

The authors declare that they have no competing interests.

## Authors' contributions

The ontological framework was conceived and developed by JH, who also prepared the manuscript. JG contributed to the development of framework and also worked extensively on manuscript preparation. AB contributed the implementation and writing for the semantic mapping module. MS provided expertise from the plant science perspective and provided guidance during manuscript preparation. LV was our resident ontology expert whose insight was critical during framework development and manuscript preparation. CRS directed the project and provided essential guidance during development of the framework as well as during preparation of the manuscript. All authors read and approved the final manuscript.

## Supplementary Material

Additional file 1 **This file contains two Excel sheets**. The first contains a listing of generated rules for the maize leaf and tomato fruit phenotypes. The second sheet has detailed information about the antecedents of each rule. Each antecedent is connected to its rule by the value in the "RuleID" column. Click here for file

Additional file 2 **This file contains the complete sample ontologies in the common format (OWL) for our constructed VPheno Ontology for maize lesion mimic mutants and our ontology of imagery and computer algorithms**. It can be loaded into Protégé for viewing. Click here for file
